# Sublethal Endpoints in Non-target Organism Testing for Insect-Active GE Crops

**DOI:** 10.3389/fbioe.2020.00556

**Published:** 2020-06-09

**Authors:** Andrew Roberts, Chad J. Boeckman, Marina Mühl, Jörg Romeis, John L. Teem, Fernando H. Valicente, Judith K. Brown, Martin G. Edwards, Steven L. Levine, Rachel L. Melnick, Thais B. Rodrigues, Ana M. Vélez, Xuguo Zhou, Richard L. Hellmich

**Affiliations:** ^1^Agriculture and Food Systems Institute, Washington, DC, United States; ^2^Corteva Agriscience^TM^, Johnston, IA, United States; ^3^Ministerio de Agricultura, Ganadería y Pesca, Dirección de Biotecnología, Buenos Aires, Argentina; ^4^Research Division Agroecology and Environment, Agroscope, Zurich, Switzerland; ^5^Embrapa Milho e Sorgo, Sete Lagoas, Switzerland; ^6^School of Plant Sciences, The University of Arizona, Tucson, AZ, United States; ^7^School of Natural and Environmental Sciences, Newcastle University, Newcastle upon Tyne, United Kingdom; ^8^Bayer Crop Science, Chesterfield, MO, United States; ^9^Greenlight Biosciences, Inc., Medford, MA, United States; ^10^Department of Entomology, University of Nebraska-Lincoln, Lincoln, NE, United States; ^11^Department of Entomology, University of Kentucky, Lexington, KY, United States; ^12^USDA, Corn Insects and Crop Genetics Research Unit, Ames, IA, United States; ^13^Department of Entomology, Iowa State University, Ames, IA, United States

**Keywords:** non-target organisms, sublethal endpoints, *Bt* Cry, GE plants, environmental risk assessment, RNAi

## Abstract

Historically, genetically engineered (GE) plants that have incorporated genes conferring insect protection have primarily used Cry proteins derived from *Bacillus thuringiensis* (*Bt*) to achieve their insecticidal phenotype. As a result, regulators have developed a level of familiarity and confidence in reviewing plants incorporating these insecticidal proteins. However, new technologies have been developed that produce GE plants that incorporate pest protection by triggering an RNA interference (RNAi) response or proteins other than *Bt* Cry proteins. These technologies have new modes of action. Although the overall assessment paradigm for GE plants is robust, there are ongoing discussions about the appropriate tests and measurement endpoints needed to inform non-target arthropod assessment for technologies that have a different mode of action than the *Bt* Cry proteins. As a result, increasing attention is being paid to the use of sublethal endpoints and their value for environmental risk assessment (ERA). This review focuses on the current status and history of sublethal endpoint use in insect-active GE crops, and evaluates the future use of sublethal endpoints for new and emerging technologies. It builds upon presentations made at the Workshop on Sublethal Endpoints for Non-target Organism Testing for Non-*Bt* GE Crops (Washington DC, USA, 4–5 March 2019), and the discussions of government, academic and industry scientists convened for the purpose of reviewing the progress and status of sublethal endpoint testing in non-target organisms.

## Introduction

**Figure 1:**
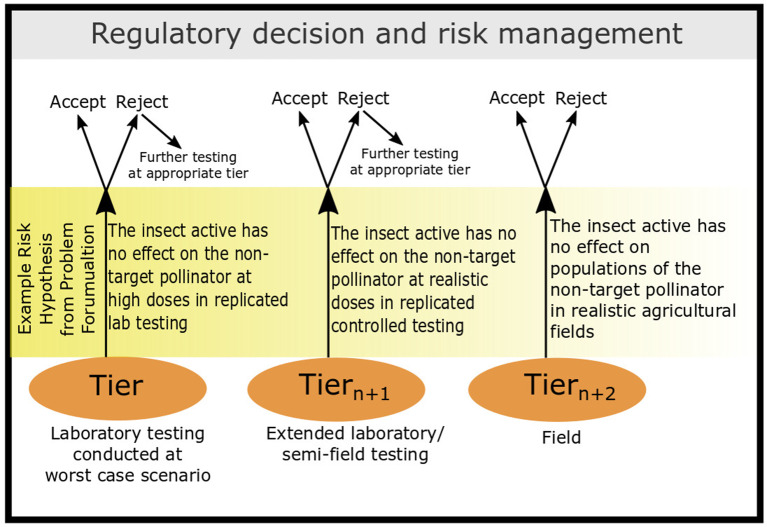
This figure details the tiered structure of testing used during the ERA process. This is hypothesis-based testing, where the initial tests are conducted at doses higher than what is encountered in a natural setting to simulate a worst-case scenario exposure. Example risk hypotheses are provided for NTOs to demonstrate how this structure can be used to test sub-lethal effects. This figure was adapted from Romeis et al. (2008).

The introduction of insect-resistant GE crops began in the 1990s, with a number of today's crops incorporating *Bt* Cry proteins (Koch et al., 2015; Naranjo et al., 2020). According to the International Service for the Acquisition of Agri-biotech Applications (ISAAA) GM Approval database^1^, 85 transformation events involving *Bt* Cry protein expressed in 10 crops received regulatory approval somewhere in the world by the end of 2019. These have been further incorporated into 206 stacks (that combine two or more GE traits) that have received additional regulatory approvals^1^. These approvals have each been accompanied by an ERA that has typically focused on identifying the target range and specificity of the *Bt* Cry proteins using a tiered approach to non-target testing that is very similar to the approach used in the assessment of chemical pesticides (Garcia-Alonso et al., 2006; Romeis et al., 2008; Figure 1). Tier-1 testing involves the use of surrogate species tested under worst-case exposure conditions in the laboratory to identify potential hazards to them. Specific endpoints, typically including mortality, are measured in tier-1 tests under conditions of exposure to concentrations, usually several fold higher than concentrations expected in the field (Romeis et al., 2008). In the absence of relevant negative effects in test species at high exposures, a conclusion that the likelihood of adverse ecological effects under realistic conditions is low or negligible can be supported (Romeis et al., 2013b). If negative effects are observed under worst-case conditions, then higher tier studies are conducted to establish if the effect is relevant under more realistic conditions (i.e., lower dose; Rose, 2007; EFSA, 2010; Figure 1).

After more than 20 years of use in the field, there is a substantial history associated with GE plants incorporating *Bt* Cry proteins and their safety in the environment (Mendelsohn et al., 2003; Naranjo, 2009; Duan et al., 2010; Center for Environmental Risk Assessment, 2012; Guo et al., 2014; Koch et al., 2016; Romeis et al., 2019). The utility of a tiered approach using mortality as the primary endpoint is supported by experience with *Bt* Cry proteins and by an understanding of the mode of action, target specificity and exposure levels for these proteins. However, new pest control technologies, including non-*Bt* Cry proteins and the use of RNAi, have led to an increased interest in sublethal endpoints testing. This interest is due to several factors, including broader interest in sublethal impacts of chemicals, differences in the mode of action, and the length of time required to observe an effect, and concerns about cumulative or additive effects of multiple stressors in the environment.

While the use of sublethal endpoints is potentially informative for the ERA of non-*Bt* GE plants, there are a number of challenges to implementing this approach. These include the wide variety of potential endpoints from which to choose and difficulties interpreting the relationship between sublethal endpoints in laboratory studies and observable effects in the field. This paper addresses some of these challenges by examining sublethal endpoints in the context of insect-resistant GE plants and considering when inclusion of sublethal endpoints may or may not be warranted in the context of problem formulation for a case-specific ERA. This review has been informed by discussions at a workshop convened by the ILSI Research Foundation (Washington, DC, USA, 4–5 March 2019) that asked participants to consider the relevance of sublethal endpoint testing using a case study approach.

## Non-Target Organism Assessment

### Problem Formulation

**Figure 2:**
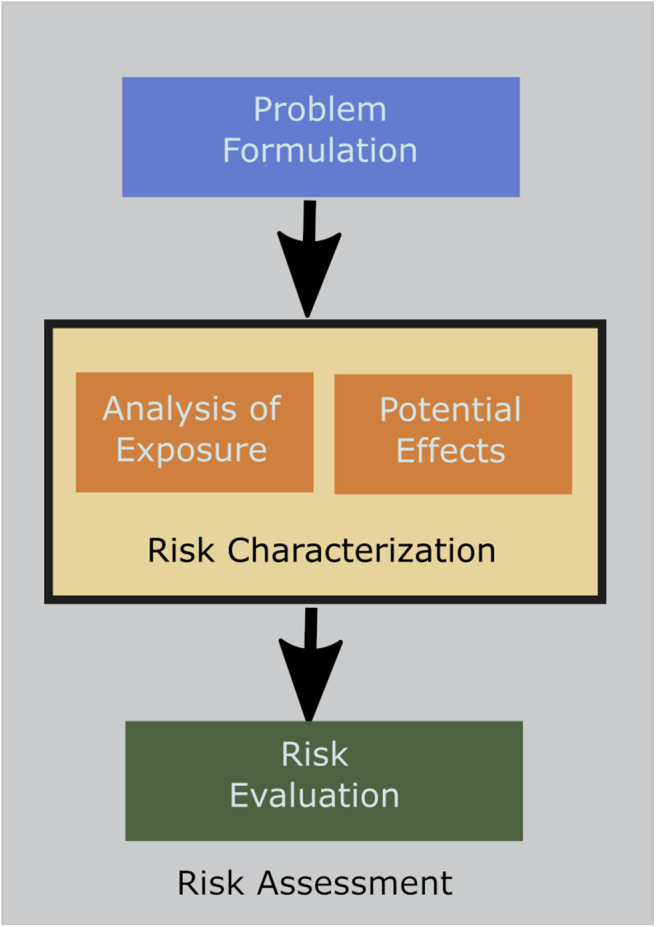
This figure provides details of the broad steps used in the risk assessment process. All risk assessments start with problem formulation to develop the hypotheses that need to be tested. Once problem formulation is completed risk characterization is conducted with analysis of exposure and testing to determine the potential effects of the exposure. Data from the risk characterization studies are used in the risk evaluation process. This figure was adapted from Wolt et al. (2010).

The process of identifying and refining the information that will be informative for case-specific ERA is referred to as problem formulation (USEPA, 2016). The mechanics of problem formulation can be described in a number of ways (Hill, 2005; OGTR, 2009; Wolt et al., 2010; Gray, 2012), but the process involves a series of steps that incorporate context for the decision being made, information about the receiving environment and the societal values or protection goals that are identified in relevant laws and regulations. Because these protection goals are often broad, case specific ERA requires the refinement of operational protection goals and the subsequent identification of assessment endpoints, which allow the testing of relevant hypotheses to inform the assessment (Sanvido et al., 2012; Devos et al., 2015). Data are then collected under laboratory, semi-field or field conditions for measurement endpoints that are related to those assessment endpoints (Garcia-Alonso and Raybould, 2014). The advantage of problem formulation is that it provides an explicit rationale for how and why a particular measurement endpoint will be informative to an ERA (Figure 2).

For most risk assessments on non-target invertebrates, operational protection goals are reliant on maintaining populations of value or beneficial arthropods that contribute to important ecosystem services (Devos et al., 2015). Among the most relevant have been populations of pollinators, parasitoids, and predators as well as charismatic, protected, threatened, or endangered species, for which an exposure assessment indicates they will have a meaningful exposure to the GE plant. Once the particular species of interest are identified, appropriate surrogate, indicator, or focal species are selected for testing (Rose, 2007; EFSA, 2010).

### NTO Study Design

As with any research or regulatory study, the design of an non-target organism (NTO) study must be appropriate for the intended end use of the data. Well-designed early-tier studies are intended to identify any hazards that require further study. Careful consideration, therefore, must be given to ensuring that tests are reliable and especially that false negative results (i.e., failing to identify a hazard) are avoided since they would lead to the release of hazardous material. False positive results, on the other hand, also should be avoided since they might have consequences, even beyond the triggering of additional studies (Romeis et al., 2013a). The first step is to identify appropriate surrogate species (Carstens et al., 2014). These should be chosen based on the representativeness of species of importance in the expected receiving environment, and ideally represent a functional group of interest. As a practical consideration, it is useful to select a surrogate from readily available laboratory reared strains. This provides uniformity to the test and control groups as well as reproducibility of results obtained from multiple laboratories. A surrogate species may additionally be selected based on its sensitivity to the test substance, taking into account what is known about the spectrum of activity of the insecticidal protein. For example, when the surrogate species and the target pests are more closely related phylogenetically, it is more probable that the surrogate species will be sensitive to the test substance (Romeis et al., 2013a). NTO tests should be designed to expose the surrogate to the test substance in a way that maximizes the likelihood of detecting an effect and represents a relevant exposure pathway (e.g., dietary exposure). While juveniles are usually expected to be more susceptible in some species, other species may instead or also have susceptible adults, so it is advantageous to test multiple life stages provided there is a reasonable exposure pathway for those life stages (Romeis et al., 2011, 2013b).

When designing the study protocol, the most expedient approach is to use an already available artificial diet into which both test substances and positive controls can be easily incorporated. This allows the concentrations of the test substance to exceed the expected environmental exposure in order to test levels that exceed worst-case exposures. If an artificial diet is not available, or not suitable for incorporation of the test substance, then GE plant material can be used. However, this may limit the test dose possibilities and reduces the margin of exposure. Furthermore, it might be a challenge to identify the appropriate non-transformed control material.

The use of appropriate controls is a critical component for ensuring that test results are reliable and meaningful (Romeis et al., 2011). When plant material is used as a test substance, near-isogenic lines are preferred as a control in order to eliminate confounding variables in the composition of the materials, unrelated to the GE trait. For NTO studies, negative controls should be designed to mimic the test conditions as closely as possible, and usually use the same test diet with an inert ingredient in place of the test substance. Negative controls also are essential to evaluate if specific *a priori* assay performance criteria were met (e.g., level of acceptable control mortality), to ensure that any observed effects are not due to nutritional inadequacies, failure of the test arthropods to eat the diet or inappropriate conditions in the experimental design for the health of the test arthropods. Similarly, positive controls are recommended to ensure that the test system is functioning as intended. This includes verifying that the test animal is consuming the test substance. To ensure this, having a positive control that closely mimics the mode of action, or at least requires the same route of exposure as the test substance is desirable. Moreover, recombinant insecticidal proteins produced in microorganisms are often used as the test substance in place of plant derived proteins because of the impracticality of purifying a sufficient quantity of protein from the plants. For this reason, it is important to assess the functional and biochemical equivalence of the test substance with the protein expressed by the GE crop (Raybould et al., 2013). Another aspect to consider is the biological activity of the test substance in the diet: a parallel bioassay is usually performed using a target species in order to check that the insecticidal protein is stable and has the expected level of biological activity in the diet. In practice, not every test system is able to meet the definition of an ideal NTO study. However, as long as the methods and protocols are reported accurately and the limitations of the study are understood and explained, these studies should still be considered in a weight-of-evidence approach. As a minimum standard, tier 1 studies should include a worst-case-exposure scenario, confirmation that the test species is exposed to the biologically active test substance, and the use of a negative experimental control (De Schrijver et al., 2016).

### Measurement Endpoints in NTO Testing

Historically, the primary and most common measurement endpoint for an early tier laboratory study is mortality. There are a number of reasons for this, including that it is normally unambiguous, easy to measure and has a clear and direct relationship to potential adverse effects on populations of NTOs and ecosystem services they provide. Because it is a common endpoint, there are study designs and methods described for measuring mortality in multiple test systems and for many test species that are validated. Adherence to these standards is often encouraged or required for submitting study results associated with regulatory reviews.

One advantage of using mortality as a study endpoint is that regulatory agencies have developed policies and practices associated with interpreting the results of mortality in NTO studies in regulatory risk assessments. A white paper generated by a panel of experts suggested 50% mortality or a 50% impact on development or weight at the maximum hazard dose in tier 1 studies with insecticidal proteins as a reasonable threshold value for determining if higher tier studies will be informative (Rose, 2007). The European Food Safety Authority (EFSA) recommends a multiplicative effect of 20% in tier 1 laboratory studies in order to trigger additional studies (EFSA, 2010). One criticism of looking at mortality as a measurement endpoint is that it may not be protective for sublethal effects that might impact populations and the ecological services provided by NTOs. While this is certainly true, it is mitigated by achieving a sufficient margin of exposure, margin of safety or other conservative features of NTO study design.

While it is often remarked that mortality is the only endpoint used in in the regulatory risk assessment of *Bt* Cry proteins (Andow and Hilbeck, 2004; Desneux and Bernal, 2010), this is not the case. As shown in Table 1, although mortality is the most common measurement endpoint, it is not unusual to see one or more sublethal endpoints recorded for a regulatory study (e.g., larval weight and development time can be collected when conducting lethality testing provided the test is of sufficient duration for the test species to reach developmental milestones). Most regulatory studies on insecticidal proteins have also recorded sublethal endpoints, but these data are not always reported in summaries or subsequent representation of the study. Sublethal endpoints that have been measured in studies for *Bt* Cry proteins include larval and adult weight, developmental timing, fecundity (number of offspring), percent completing adult development and even mobility. What is equally apparent from Table 1 is that while sublethal endpoints have been measured, sometimes it is not immediately obvious how or why particular data on sublethal endpoints are collected, and there is little consistency in how those endpoints are reported in the literature. This is not unexpected as these measurements are selected and coordinated by individual product managers in the absence of specific data requirements. Standardized and validated test protocols used to assess foliar applications of pesticides published by the International Organization for Biological and Integrated Control of Noxious Animals and Weeds (IOBC) include methods for a total of nine beneficial species (two parasitoids, seven predators) and can inform NTO testing for GE plants. With the exception of one beetle species (*Aleochara bilineata*, Coleoptera: Staphylinidae), all IOBC protocols assess mortality as an endpoint (Candolfi et al., 2000). In addition, the tests also considered sublethal endpoints, mainly based on reproduction but also food consumption and behavior of the organism.

**Table 1:** Laboratory studies with beneficial non-target invertebrates (predators, parasitoids, pollinators) or surrogate species for the soil and aquatic environment to support the regulatory risk assessments of plant expressed insecticidal *Bt* Cry proteins.

**Species**	**Life-stage exposed**	**Measurement endpoints**	**References**
**Predators**			
*Aleochara bilineata*	Adults	Fecundity, offspring survival	Stacey et al., 2006^a^
		Fecundity	Raybould and Vlachos, 2011
*Coccinella septempunctata*	Larvae	Mortality, development time, adult weight	De Schrijver et al., 2016^b^
	Larvae, adults	Larval mortality, development time, adult mortality	Stacey et al., 2006^a^;
		Development time, adult weight, fecundity, fertility	De Schrijver et al., 2016^b^
	Adults	Mortality	Raybould and Vlachos, 2011
*Coleomegilla maculata*	Larvae	Mortality, development time, adult weight	Duan et al., 2002; Devos et al., 2012^c^; De Schrijver et al., 2016^b^; Bachman et al., 2017
		Mortality, weight	De Schrijver et al., 2016^b^
	Adults	Mortality, adult weight fecundity	Duan et al., 2002
		Mortality	Raybould and Vlachos, 2011; Devos et al., 2012^c^
*Hippodamia convergens*	Adults	Mortality	Devos et al., 2012^c^; De Schrijver et al., 2016^b^
*Poecilus chalcites*	Larvae	Mortality, development time, development rate, weight	Duan et al., 2006
*Poecilus cupreus*	Larvae	Mortality, adult weight	Stacey et al., 2006^a^
		Mortality, Development time, adult weight	De Schrijver et al., 2016^b^
*Orius insidiosus*	Nymphs	Mortality, percent developing into adults	Stacey et al., 2006; Duan et al., 2008^a^; Bachman et al., 2017
		Mortality	Raybould and Vlachos, 2011; De Schrijver et al., 2016^b^
*Orius laevigatus*	Nymphs	Mortality, development time	De Schrijver et al., 2016^b^
*Chrysoperla carnea*	Larvae	Mortality	Raybould and Vlachos, 2011; Devos et al., 2012^c^; De Schrijver et al., 2016^b^
**Parasitoids**			
*Pediobius foveolatus*	Adults	Mortality	Bachman et al., 2017
*Nasonia vitripennis*	Adults	Mortality	Devos et al., 2012^c^; De Schrijver et al., 2016^b^
**Pollinator**			
*Apis mellifera*	Larvae	Mortality	Duan et al., 2008; Raybould and Vlachos, 2011; Devos et al., 2012; Bachman et al., 2017^c^
		Mortality, development time	Devos et al., 2012^c^; De Schrijver et al., 2016^b^
		Brood development	Raybould et al., 2007
	Adults	Mortality	Duan et al., 2008; Devos et al., 2012^c^
**Soil organism**			
*Folsomia candida*	Juveniles	Mortality, number of offspring	Raybould and Vlachos, 2011; Devos et al., 2012^c^; De Schrijver et al., 2016^b^; Bachman et al., 2017
**Aquatic organisms**			
*Daphnia magna*	Juveniles	Mortality	Devos et al., 2012^c^
		Mobility	De Schrijver et al., 2016^b^
*Culex quinquefasciatus*	Larvae	Mortality	De Schrijver et al., 2016^b^

*This table only represents sublethal endpoint data collected as part of a regulatory study and does not present sublethal endpoint data collected as part of solely academic studies*.

a *Data provided in Stacey et al. (2006) are also listed in Raybould et al. (2007). Raybould et al. (2007) summarizes the data that were submitted to the regulatory authority. Interestingly, for all but one of the organisms tested (O. insidiosus; P. cupreus; and A. bilineata) not all sublethal endpoint measured by Stacey et al. (2006) are also reported by Raybould (2007)*.

b *De Schrijver et al. (2016) lists unpublished data from regulatory studies*.

c *Devos et al. (2012) lists unpublished data from regulatory studies*.

The selection of measurement endpoints associated with NTO assessment should be guided by a proper problem formulation that incorporates case-specific information pertinent to the assessment. In this way, measurement endpoints are selected that are clearly linked to an assessment endpoint and associated with the risk hypothesis. Risk hypotheses should not be generic, but rather case-specific and informed by what is known about the insect active routes of exposure and its effects on sensitive or target species. Also, prior to the study, the project managers and risk assessors should know specifically how the measurement endpoints will be interpreted and how they will be used in the decision process. If it cannot be clearly articulated how study results will inform the assessment, then the measurement endpoints for the study may not be well-aligned with protection goals and decision-making priorities. Finally, the measurement endpoints should be designed to incorporate the practical realities and limitations associated with available and appropriate relevant test species.

## Experience With Sublethal Endpoint Testing in Non-*Bt* GE Crops

The development of novel, non-*Bt* Cry proteins for insect control and of non-protein based methods such as RNAi has been accompanied by an increased interest in assessing sublethal impacts. Publications related to these new technologies demonstrate some of the ways that sublethal endpoints are used.

### RNAi and MON87411

The potential use of RNAi for pest control has been widely discussed (Burand and Hunter, 2013) and regulators are considering the use of RNAi to control insect pest in relation to risk assessment (USEPA, 2014; Casacuberta et al., 2015; Roberts et al., 2015). Presently, a single insect protected crop using RNAi as a mechanism has been approved for commercialization. The effect of the RNAi in the target pest, the western corn rootworm (WCR, *Diabrotica virgifera virgifera*), is triggered by the presence of a dsRNA targeting a housekeeping gene. MON87411 targets the WCR *Snf7* gene and its mode of action has been well-characterized (Bolognesi et al., 2012; Ramaseshadri et al., 2013). Uptake of *DvSnf7* RNA generates eventual mortality or severe growth inhibition, which can be observed 5 days after exposure to the test substance. There is also a relationship between exposure duration and eventual growth inhibition at lower doses, but short exposures at high doses are sufficient to induce mortality (Bolognesi et al., 2012). A description of the published ERA for MON87411 expressing *DvSnf7* RNA, includes a description of NTO testing conducted in support of the risk assessment (USEPA, 2015; Bachman et al., 2016). A range of vertebrate tests were conducted, as well as arthropod testing including toxicity assays for seven species of arthropod. In addition to mortality, sublethal endpoints were observed for each species and included measures of time to adulthood, percent adult emergence, adult biomass (weight), and fecundity (number of surviving offspring produced). Tests were conducted with concentrations of the test substance in excess of 10-fold the maximum expected environmental concentration and the duration of the test period was in excess of the time required to see an effect in the target species. The selection of surrogate species took into account the mode of action, considering that coleopterans (beetles) show significantly greater sensitivity to ingested dsRNA than other arthropod orders (Roberts et al., 2015). Because this order of insect includes the target species, WCR, it provides a good illustration of how phylogenetic relationships and an understanding of the mode of action can facilitate the choice of surrogates. Additionally, bioinformatics was used as a complementary tool to perform a screening and to identify potential surrogate species based upon the presence of sequence matches. Sequence alignment between the genome and *DvSnf7* informed the number and type of species tested, focusing on those which were considered most likely to be informative (Bachman et al., 2016).

### Non-*Bt* Cry Insecticidal Proteins: IPD072Aa

One example of a non-*Bt* insecticidal protein that has been subject to extensive NTO assessment is IPD072Aa, isolated from *Pseudomonas chlororaphis*, which has activity against WCR (Schellenberger et al., 2016). The IPD072Aa protein has been the subject of bioassays to determine the spectrum of activity in order to inform the NTO risk assessment (Boeckman et al., 2019). As the target pest is a coleopteran, bioassays were conducted with 11 species of Coleoptera, representing four families. Additionally, four species of lepidopteran (moths and butterflies) representing four families in this order were tested. Measurement endpoints included mortality as well as weight for all but one species tested, and time to emergence for two species of ladybird beetles. No observed effects were reported for any of the Lepidoptera species, but both mortality and sublethal effects were observed at varying protein concentrations in some of the tested Coleoptera. In line with best practices for NTO study design (see NTO Study Design above) the criteria for selection of species to characterize the spectrum of activity of IPD072Aa was based on several factors such as the phylogenetic relationship between the species and WCR, established laboratory bioassay methodologies, availability of laboratory reared insects, a known suitable diet and reproducibility of the measurement endpoints (Boeckman et al., 2019).

IPD072Aa has a midgut site of action (SOA) where it targets and disrupts midgut epithelial cells causing breakdown of the epithelial lining in WCR through what appears to be a non-pore forming mechanism (Schellenberger et al., 2016). The ability of IPD072Aa to kill WCR larvae resistant to mCry3A or Cry34Ab1/Cry35Ab1 indicates that its target site differs from those of the *Bt* Cry proteins (Carlson et al., 2019). This knowledge related to its mode of action and the phylogentic relationship between the candidate surrogate species and WCR will guide selection of the appropriate surrogate species and measurement endpoints in NTO assays.

### Non-Cry Insecticidal Proteins: Vip3A

Vip3A is a *Bt* vegetative insecticidal protein that is active against lepidopteran pests. It has a different mode of action from Cry proteins, and when delivered as a combined treatment it has the potential to delay the evolution of pest resistance to *Bt* crops (Lee et al., 2003). A description of the ERA for MIR162, a maize event expressing the Vip3A protein, has been published (Raybould and Vlachos, 2011). The bioassays were conducted using species representing functional groups of foliar arthropods, soil-dwelling invertebrates, pollinators, wild birds and mammals, aquatic invertebrates and farmed or wild fish (Raybould and Vlachos, 2011). In addition to mortality, sublethal endpoints such as fecundity, weight increase, adult emergence, body weight, and length were recorded for some of the species tested. Depending on the species, worst-case, or conservative maximum expected environmental concentrations were used.

## Discussion

### Sublethal Endpoints Are Addressed in the Context of Existing Frameworks for NTO Assessment

The rationale for conducting NTO studies in support of regulatory risk assessments is not dependent on whether the studies are designed to measure mortality or sublethal endpoints. Before a study is conducted, the problem formulation process should identify a set of informative tests based on the pathways of environmental exposure, and an identification of relevant taxa and functional groups for which risk should be assessed. Once the appropriate tests are identified, consideration can be given to what sort of measurement endpoints will best inform the assessment. When making these decisions, it is important to keep in mind the practical limitation associated with NTO testing and risk assessment.

### Absence of a Single Definitive Test or Sublethal Endpoint

As with other types of testing, there is no single definitive test that can address every possibility of sublethal effects. As is typical of the regulatory risk assessment process, consideration of whether a test is necessary should be done on a case-by-case basis, focusing on the value of the information for reaching conclusions about overall risk. Although flexibility in testing allows sublethal tests to be tailored to specific needs of a chosen test system, non-uniformity in testing procedures can make it difficult to compare results obtained by independent researchers conducting experiments and then reporting sublethal impacts in the literature. These comparisons may provide helpful context for regulatory considerations. The endpoints that have been previously utilized to measure sublethal effects for all types of insect-active GE plants (i.e., weight, growth and developmental time, fecundity) appear to be useful and sufficient for sublethal testing of non-*Bt* Cry insect actives, both protein-based and dsRNA-based.

### Effect of the Active on the Target Can Indicate the Utility of Sublethal Endpoints in NTO Testing

Knowledge regarding the mode of action and time to effect, as it relates to the effect of the insect active on the target insect, is instructive for ascertaining whether sublethal endpoint testing is likely warranted in NTOs. For example, if an insect active targets a cellular process that broadly affects growth (e.g., protein synthesis) it may be reasonably expected that collection of data measuring sublethal endpoints such as weight, development time, and reproduction may be warranted. If those endpoints are affected in the target organism, they may also be affected in NTOs. Additionally, the time that it takes for sublethal effects to manifest in the target organism during lethality testing may also be an indication that sublethal endpoint testing is warranted for NTO assessment. If mortality in the target organism is delayed, but indications of mortality are evident earlier due to the onset of sublethal effects such as delayed growth or development, then it may make sense to look for these effects in NTO assessment as well.

### Sublethal Endpoints Need to Relate to Ecologically Relevant Assessment Endpoints

Sublethal endpoints that relate to measurable ecologically relevant endpoints will be much easier to interpret than more complicated endpoints. Typically, measurement endpoints for sublethal effects that include development time, growth/weight and reproduction are used for data collection, and these endpoints are readily quantifiable and relatable to assessment endpoints such as population size. While a variety of additional sublethal endpoints reflecting more ambiguous measurements have been reported in the published literature (i.e., feeding behavior and learning performance; Ramirez-Romero et al., 2008), the interpretation of these endpoints with respect to ecological outcomes is challenging.

### Practical Considerations for Developing New Test Systems

Any NTO testing is dependent on the existence or the development of a well-characterized and validated test system. Some existing test systems may lend themselves to the collection of sublethal endpoint data, but others may not. Thus, development of new test systems may be required when and if the problem formulation indicates that a particular insect active is likely to cause sublethal effects in relevant NTOs. In either case, before the tests are conducted it is important to assure that the results of the sublethal measurements will be meaningful for risk assessment.

### Addressing Knowledge Deficits

For RNAi-based insect actives, bioinformatics will become increasingly important in predicting potential adverse effects associated with exposure to dsRNA. However, there are currently significant gaps in bioinformatics data for both pest organisms and for ecologically important taxonomic groups, as well as a lack of information on which taxonomic groups are (in)sensitive to environmental RNAi. Narrowing those information gaps will provide a better understanding of the extent to which different NTOs need to be assessed for a case-specific assessment for a particular environmental RNAi.

More sublethal endpoint data are collected than is widely acknowledged. In part, this is, due to the primary status of mortality in testing guidelines and in summary reports or journal publications where sublethal endpoint measurements often are relegated to supplementary material. Finding new means of sharing this information (and improving access to it) is needed in the future to increase the potential usefulness of this information for risk assessments. Similarly, data collected during early characterization of pesticidal proteins may be considered proprietary in nature, presenting a barrier to broad distribution. Whenever possible, however, mechanisms should be encouraged to improve access to this type of information. There are several recent examples where early characterization of pesticidal molecules have been published (Bachman et al., 2013, 2017; Boeckman et al., 2019). However, the research community is encouraged to find new means of storing and disseminating information that often is omitted from peer-reviewed publications but has value as a collective resource in further development of NTO risk assessment methodology.

## Conclusions

NTO testing is conducted in support of regulatory decision making, and therefore must be designed for this purpose, rather than simply to conduct scientifically interesting experiments (Raybould, 2010). The testing associated with any particular insect-active GE crop should be informed by a problem formulation process. Problem formulation takes into account what is known about the insect-active protein, the crop, and the expected interactions between NTOs and the associated insect-active crop, as well as the availability of well-developed test systems that facilitate the interpretation of test results in a regulatory context. The problem formulation process remains fundamentally the same whether the measurement endpoint is mortality or sublethal endpoints. When selecting sublethal endpoints for consideration, a risk hypothesis should link the sublethal endpoint to an assessment endpoint and the associated protection goal. Because most NTOs are protected at the population level and NTO communities at the functional level, typically, sublethal endpoints that are related to growth, development, and reproduction and which can be easily extrapolated to population level effects are most informative. While additional sublethal endpoints might be measurable, they should only be considered for regulatory testing if there is a clear relationship to a protection goal and the results are likely to reduce uncertainties associated with the NTO assessment. A review of recent and past measurement of sublethal endpoints collected to inform regulatory studies of plant incorporated insecticidal *Bt* Cry proteins is summarized in Table 1. These data suggest sublethal endpoints for current insect resistant GE crops are observed and measured more routinely than is often claimed in the literature.

A long history of standardization exists that can inform the future of NTO testing. While standard methods are not absolutely required for testing possible sublethal impacts, such studies can be informative for risk assessment. However, for these studies to be informative, there should be a clear understanding of what data are being collected and what is the rationale for collecting them. When published in peer reviewed publications, these sublethal endpoints are often published as supplemental data. If sublethal testing is done, and data are reported, these data should be presented more prominently in research reports. This practice would promote a broader understanding and further discussion of the utility of sublethal endpoints and enhance their usefulness to the risk assessment process.

## Disclosure

The information and views are those of the authors as individuals and experts in the field, and do not necessarily represent those of the organizations where they work. Mention of a proprietary product does not constitute an endorsement or a recommendation for its use by Greenlight Biosciences, Inc., Iowa State University, ILSI Research Foundation, Newcastle University, The University of Arizona, University of Kentucky, University of Nebraska-Lincoln, or USDA.

## Author Contributions

JR, CB, RH, MM, FV, and AR lead the initial drafting of the manuscript. CB, JB, ME, RH, SL, MM, RM, JT, TR, AV, JR, FV, XZ, and AR all made significant contributions to the drafting of the article and developing the table. CB, JB, ME, RH, SL, MM, TR, AV, JR, FV, XZ, and AR attended the ABSTC sponsored workshop. JT organized the ABSTC sponsored workshop. RM, AV, and AR developed and created the figures. RM and AR lead the editorial efforts to develop the manuscript.

## Conflict of Interest

CB is employed by Corteva Agriscience, SL is employed by Bayer Crop Science, and TR is employed by Greenlight Biosciences, Inc. AV has received funding from Bayer Crop Science and Corteva Agriscience to perform research on RNAi for insect pest management and has six issued RNAi patents. The remaining authors declare that the research was conducted in the absence of any commercial or financial relationships that could be construed as a potential conflict of interest.

## Footnotes

^1^http://www.isaaa.org/gmapprovaldatabase/
